# Impacts of mutation effects and population size on mutation rate in asexual populations: a simulation study

**DOI:** 10.1186/1471-2148-10-298

**Published:** 2010-09-30

**Authors:** Xiaoqian Jiang, Baolin Mu, Zhuoran Huang, Mingjing Zhang, Xiaojuan Wang, Shiheng Tao

**Affiliations:** 1 Bioinformatics center, Northwest A&F University, Yangling, Shaanxi, 712100, China; 2 College of Life Science, Northwest A&F University, Yangling, Shaanxi, 712100, China; 3 College of Information Engineering, Northwest A&F University, Yangling, Shaanxi, 712100, China

## Abstract

**Background:**

In any natural population, mutation is the primary source of genetic variation required for evolutionary novelty and adaptation. Nevertheless, most mutations, especially those with phenotypic effects, are harmful and are consequently removed by natural selection. For this reason, under natural selection, an organism will evolve to a lower mutation rate. Overall, the action of natural selection on mutation rate is related to population size and mutation effects. Although theoretical work has intensively investigated the relationship between natural selection and mutation rate, most of these studies have focused on individual competition within a population, rather than on competition among populations. The aim of the present study was to use computer simulations to investigate how natural selection adjusts mutation rate among asexually reproducing subpopulations with different mutation rates.

**Results:**

The competition results for the different subpopulations showed that a population could evolve to an "optimum" mutation rate during long-term evolution, and that this rate was modulated by both population size and mutation effects. A larger population could evolve to a higher optimum mutation rate than could a smaller population. The optimum mutation rate depended on both the fraction and the effects of beneficial mutations, rather than on the effects of deleterious ones. The optimum mutation rate increased with either the fraction or the effects of beneficial mutations. When strongly favored mutations appeared, the optimum mutation rate was elevated to a much higher level. The competition time among the subpopulations also substantially shortened.

**Conclusions:**

Competition at the population level revealed that the evolution of the mutation rate in asexual populations was determined by both population size and mutation effects. The most striking finding was that beneficial mutations, rather than deleterious mutations, were the leading force that modulated the optimum mutation rate. The initial configuration of the population appeared to have no effect on these conclusions, confirming the robustness of the simulation method developed in the present study. These findings might further explain the lower mutation rates observed in most asexual organisms, as well as the higher mutation rates in some viruses.

## Background

Understanding the genetic structure of populations requires knowledge of the mutation rate, an important parameter of evolution. One of the essential problems in population genetics is determining how natural selection acts on the mutation rate of an organism during long-term evolution. Although mutation provides the ultimate source of genetic variation, it typically leads to decreased fitness. Even when a population is in the process of adaptation, the majority of its mutations are still deleterious and will ultimately be eliminated by selection. This type of selection pressure was first observed by Sturtevant [1], who questioned why the mutation rates never fall to zero.

Since Sturtevant's pioneering work, the evolution of mutation rate has been researched by many evolutionary biologists and our understanding of this question has been improved in many respects. At present, several methods have been proposed for characterization of the evolution of mutation rate, including direct estimates from mutation accumulation experiments [2-5], indirect estimates from comparisons of DNA sequences among related species [6-8], and theoretical analysis [9-12]. Overall, these methods have been successful in detecting and estimating mutation rates, as well as in describing the relationship between natural selection and mutation rate. Drake [13] suggested that the genome mutation rate (*U*) in DNA-based microbes was about 0.0034 per generation despite a wide variation in genome size. This relatively constant observed value indicates that the genome mutation rate in microbes has evolved perfectly to fit the pace of environmental changes through natural selection. Several theoretical methods have the potential to explain Drake's observation from different perspectives [11,12,14,15]. For instance, a previous classical research on the evolution of mutation rate was investigated by Leigh based on mathematical analysis [11]. He described the long-term fate of a modifier in infinite asexual populations, and showed that the error rate of DNA replication was exactly equal to the rate of environmental changes. Orr [15] found that the optimum mutation rate was equal to the harmonic mean of the selection coefficients of deleterious mutations when selection for beneficial mutations was assumed milder than selection for deleterious mutations. However, most theoretical analysis methods have focused solely on individual competition within a population. Competition among populations has not yet been sufficiently investigated with respect to the evolution of mutation rates.

In any finite population, the process of evolution is well known to be influenced by population size and mutation effects [16]. Beneficial mutations are more frequently fixed in large populations than in small ones, whereas deleterious mutations are more frequently eliminated. Two studies, one based on a theoretical mathematical model [17], and one on experiments of digital organisms [18], arrived at a similar conclusion; namely, that mutational robustness tended to decline with increasing population size, and thus selection in small populations would favor robustness mechanisms. In a population of a given size, the process of evolution will depend on the relative rate of appearance of deleterious and beneficial mutations as well as their actual mutational effects. Selection associated with deleterious mutations will favor lower mutation rates, while beneficial mutations will favor higher mutation rates [9]. Nevertheless, the evolution of extremely high mutation rates is unlikely to occur unless organisms are under special circumstances [19] for the reason that beneficial mutations rarely compensate for deleterious mutations. The importance of this interplay between mutation rate and its effects was pointed out by Keightley [20], who showed that the genome-wide mutation rate and the distribution of fitness effects of mutations could not be simultaneously estimated because they are confounded with one another: a high mutation rate can usually be explained by a low variance in fitness effects, or a low mutation rate with a high variance in fitness effects. Unfortunately, this conclusion is true only for deleterious mutations and further investigation is needed for cases where beneficial mutations also occur.

When both deleterious and beneficial mutations are present, it is necessary to explore whether an organism could evolve to an "optimum" mutation rate under these two opposing forces. The nature of the dominating factors that shape the optimum mutation rate also needs to be determined. In the present paper, we have developed a simulation method based on competition among subpopulations with different mutation rates to examine how selection may impact the evolution of genome mutation rate. Our results indicate that a larger population could tolerate a higher mutation rate than could a smaller one. The optimum mutation rate depends almost exclusively on the effects of beneficial mutations regardless of the extent of deleterious mutation effects. Possible reasons for these findings are discussed in comparison with previous studies.

## Methods

### The model

We consider a finite strictly asexual haploid population (with constant population size *N*) that comprises 10 subpopulations, each of which has *N/*10 individuals and a different mutation rate, with everything else equal. The rationale of the method is that these subpopulations compete for existence under natural selection and random drift. At the end of a simulation, only one subpopulation remains and the rest are extinct. The mutation rate of the remaining population becomes the "fixed" mutation rate in that competition. By simulating the process many times, we can define the most frequently fixed mutation rate as the "optimum" mutation rate.

Each of the ten subpopulations is assigned with a distinct mutation rate per genome per generation (see parameters). Both deleterious and beneficial mutations occur in each subpopulation with fractions for beneficial and deleterious mutations represented by *p_b _*and *p_d _*(i.e. 1- *p_b_*), respectively. The effects (selection coefficients) of both beneficial and deleterious mutations are drawn from continuous probability distributions. We denote *s_b _*as the effects of beneficial mutations (in which case fitness *w *is increased by a factor 1+ *s_b_*), while *s_d _*represents the effects of deleterious mutations (in which case fitness *w *is decreased by a factor 1- *s_d_*)[21].

We assume that *s_b _*follows an exponential distribution:$f(s_{b},\lambda )=\lambda e^{-\lambda s_{b}}$ with 1/λ as the mean value of the distribution. This assumption has good theoretical support from extreme-value theory and has been widely used in population genetics models [22-24]. The effects of deleterious mutations may be complex and no general assumption yet exists about the distribution of *s_d _*in analytical calculations; however, empirical studies support a gamma distribution with shape parameter smaller than one (other distributions are not necessarily excluded)[25,26]. In the present study, we assume that *s_d _*follows a skewed gamma distribution $f(s_{d},\alpha ,\beta )=s_{d}{}^{\alpha -1}e^{-s_{d}/\beta }/(\beta^{\alpha }\Gamma (\alpha ))$ (α≤1). The gamma distribution used in our simulations is truncated with the value 1.0, which is necessary to avoid producing a negative fitness. In addition, we assume that the mean effects of beneficial mutations ($\bar{s_{b}}$) are much smaller than the mean effects of deleterious ones ($\bar{s_{d}}$), which seems to be reasonable in most cases [27,28].

### Parameters

**Figure 1:**
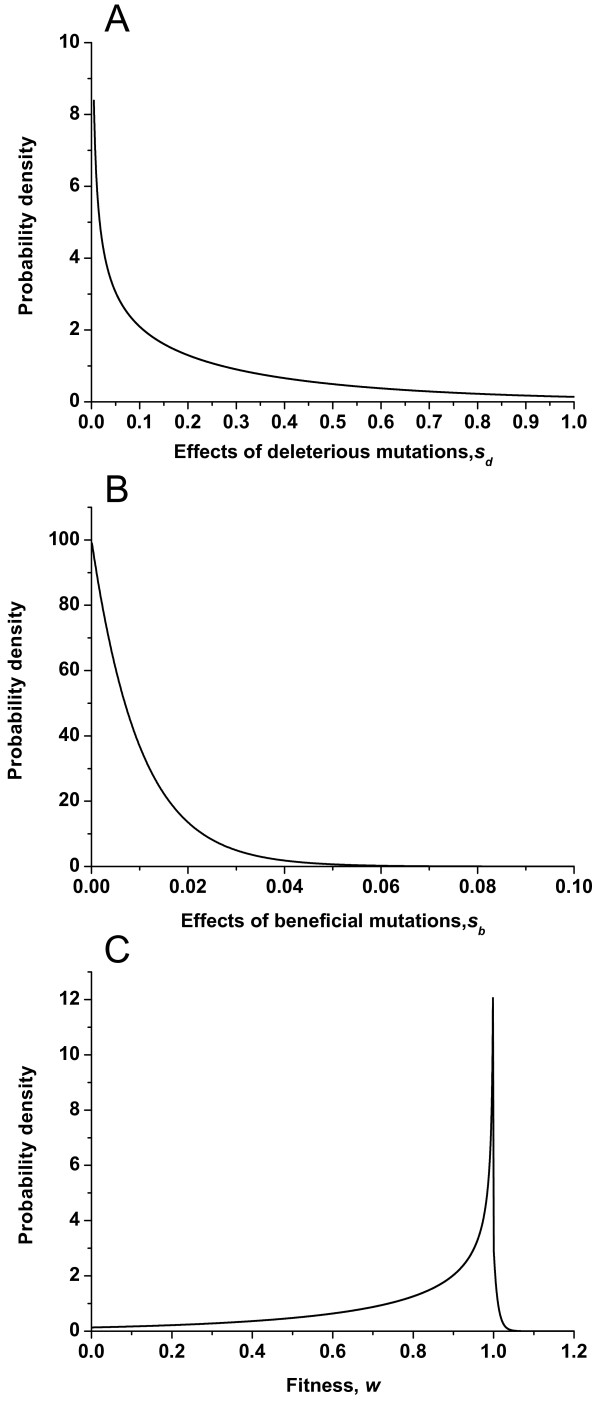
**One example of distribution of mutation effects**. (A) The effects of deleterious mutations follow a gamma distribution with *α *= 0.6 (shape parameter), *β *= 0.5 (scale parameter) and the mean effects is ${\bar{s}}_{d}=0.3$. (B) The effects of beneficial mutations follow an exponential distribution with *λ *= 100 and the mean effects is ${\bar{s}}_{b}=0.01$. (C) The distribution of fitness changes by both deleterious and beneficial mutations with *p_d _*= 97% and *p_b _*= 3%.

In our simulations, the sizes of fractions and effects of both beneficial and deleterious mutations are the most important quantitative parameters. Numerous experimental studies on microbes have shed some light on this area and some estimates of these parameters are summarized in Table 1[29-35]. These data provide the best available assumptions of parameters used in the simulations. One example of the distribution of mutation effects and the corresponding fitness variation caused by mutations we adopt is shown in Figure 1. Another essential parameter involved in the simulations is the mutation rates initially assigned to the ten subpopulations. And the logarithmic form of the mutation rates (lg(*U*)) is roughly uniformly distributed between -4 and -1. In addition, we adopt several ranges consisting of different mutation rates, which are shown in Table 2, to see if this initial range influences the optimum mutation rate.

**Table 1:** Some estimates of mutation parameters from previous experiments on microbes

	Deleterious		Beneficial		
			
Taxon	*p_d_*	$\bar{s_{d}}$	*p_b_*	$\bar{s_{b}}$	Reference
*Vesicular stomatitis virus*	29.2%	0.24	4.2%	0.042	Sanjuan *et al*. (2004)^a^[29]
*Tobacco etch potyvirus*	36.4%	0.41	0	0	Carrasco *et al*. (2007)^b^[30]
*S. cerevisiae*	-	0.22	-	-	Zeyl and DeVisser (2001)[2]
*Diploid S. cerevisiae*	-	-	5.75%	0.061	Joseph and Hall (2004)[31]
*E. coli*	-	-	-	0.02	Imhof and Schlotterer (2001)[32]
*E. coli*	-	-	-	0.054	Hegreness *et al*. (2006)[33]
*E. coli*	-	-	0.67%^c^	0.01	Perfeito *et al*. (2007)[34]
*P. fluorescens*	-	-	-	0.023~0.089	Kassen and Bataillon (2006)[35]

a, b: in both studies, *p_d _*does not include lethal mutations.

c: *p_b _*is 1/150, compared to Drake's famous genome mutation rate 0.0034[13].

**Table 2:** Summary of different mutation rates assigned initially to ten subpopulations

		Subpopulation									
		
*R_u_*		1	2	3	4	5	6	7	8	9	10
1	*U*	0.0001	0.0003	0.001	0.003	0.01	0.02	0.03	0.04	0.06	0.1
	lg*U*	-4.0	-3.5	-3.0	-2.5	-2.0	-1.7	-1.5	-1.4	-1.2	-1.0
											
2	*U*	0.0001	0.0003	0.001	0.002	0.004	0.006	0.01	0.02	0.05	0.1
	lg*U*	-4.0	-3.5	-3.0	-2.7	-2.4	-2.2	-2.0	-1.7	-1.3	-1.0
											
3	*U*	0.0001	0.0002	0.0005	0.001	0.002	0.005	0.01	0.02	0.05	0.1
	lg*U*	-4.0	-3.7	-3.3	-3.0	-2.7	-2.3	-2.0	-1.7	-1.3	-1.0
											
4	*U*	0.0002	0.0005	0.001	0.002	0.005	0.01	0.02	0.05	0.1	0.2
	lg*U*	-3.7	-3.3	-3.0	-2.7	-2.3	-2.0	-1.7	-1.3	-1.0	-0.7
											
5	*U*	0.00005	0.0001	0.0005	0.001	0.005	0.01	0.05	0.1	0.5	1
	lg*U*	-4.3	-4.0	-3.3	-3.0	-2.3	-2.0	-1.3	-1.0	-0.3	0.0

### Numerical Simulations

Throughout the study, we assume that generations are discrete and non-overlapping. In each generation, the number of new mutations (*m*) appearing in an individual belonging to the *i-th *subpopulation is drawn from a Poisson distribution $p(m,U_{i})=U_{i}{}^{m}e^{-U_{i}{}_{i}}/m!$, where *U_i _*is the genome mutation rate of the *i-th *subpopulation. The deleterious mutation rate is then given by *U_i_*×*p_d _*and the beneficial mutation rate is *U_i_*×*p_b_*. Given that a deleterious (or beneficial) mutation occurs, the fitness *w *of the individual is decreased (or increased) by 1- *s_d _*(or 1+ *s_b_*), where *s_d _*(or *s_b_*) is randomly drawn from a gamma (or exponential) distribution. Here, we assume that no epistasis occurs; therefore, all mutations have independent effects on fitness and act multiplicatively. It is possible that an individual may carry multiple mutations within a single generation. In this case, the fitness of an individual in the *n-th *generation (*w_n_*) is a function of the mutation numbers the individual carries (*m*), their mutation effects (*s_j_*), and the fitness of its parent in the (*n-1*)-*th *generation (*w_n-1_*). This function can be described as

$$w_{n}=w_{n-1}\times \prod_{j=1}^{m}\left(1\pm s_{j}\right)$$

Offspring are sampled with repetition according to a multinomial distribution, weighted by the fitness of their respective parent. We label each offspring with a unique identifier for its particular subpopulation.

We trace the numbers of individuals of each subpopulation until the population size of one subpopulation reaches *N *and the sizes of other subpopulations become zero. At this point, the process is stopped and the corresponding mutation rate of the remaining subpopulation is recorded. In addition, the number of generations one competition takes is also traced. We run simulations that vary both the population size and the mutation effects to evaluate how and to what extent these influence the competition results (see Results). Some initial conditions of the population are also relaxed to test the robustness of the method (see Discussion).

## Results

Our extensive simulations were designed to test whether natural selection could shape the optimum mutation rate, given the initial configuration of the population. Each curve represents one simulation result with 300 competitions among all the figures and each point represents the frequency of being fixed of the corresponding mutation rate. For convenience of description, we used the symbol *U_opt _*as the optimum mutation rate and *G *as the mean number of generations required for competition in one simulation. We also used *R_u _*to represent which group of mutation rates was adopted in Table 2. The simulation results suggested that the distribution of the frequencies of the fixed mutation rates was similar to a bell shape, revealing that the optimum mutation rate will be maintained within an intermediate range under natural selection rather than be kept at a minimal one.

**Figure 2:**
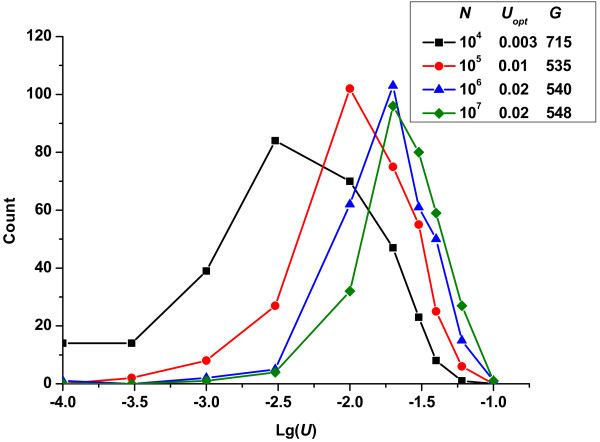
**Change in the frequencies of fixed mutation rates *vs*. lg(*U*)-given different population size *N *(*R_u _*= 1)**. In all cases, the conditions were constant: *p_b _*= 3%, ${\bar{s}}_{d}=0.3$, ${\bar{s}}_{b}=0.01$.

Figure 2 shows the change in the frequencies of fixed mutation rates with population size (*N*). Clearly, *N *had a significant effect on the frequencies of fixed mutation rates. With all other factors held constant, increasing in *N *caused the curve to shift to the right, which demonstrated that a larger population tolerated a higher mutation rate. Large populations could benefit from relatively higher mutation rates because beneficial mutations appeared more frequently and selection was more efficient in removing deleterious mutations in large populations than in small ones [36]. As Figure 2 shows, the optimum mutation rate increased from 0.003 to 0.02 when the population size varied from 10^4 ^to 10^7^.

**Figure 3:**
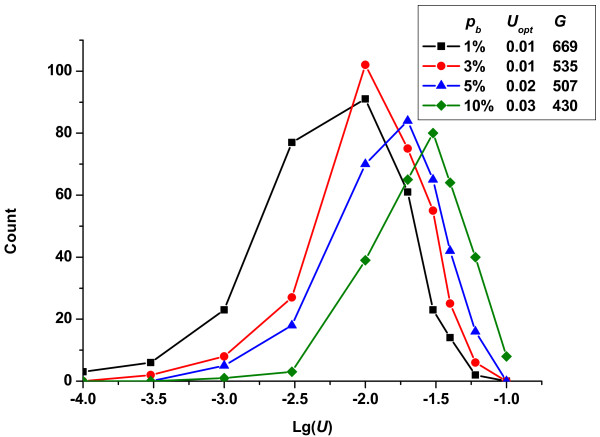
**Change in the frequencies of fixed mutation rates *vs*. lg(*U*)-given different fraction of beneficial mutations *p_b _*(*R_u _*= 1)**. In all cases, the conditions were constant: *N *= 10^5^, ${\bar{s}}_{d}=0.3$, ${\bar{s}}_{b}=0.01$.

To investigate the influence of the relative fraction of deleterious and beneficial mutations, and the combined effects of both, on the optimum mutation rate, we ran simulations where one factor was controlled and the other was varied. In Figure 3, we show the change in the frequencies of fixed mutation rates with the fraction of beneficial mutations (*p_b_*), while holding the effects of both deleterious and beneficial mutations constant. The curve was shifted to the right with increasing *p_b_*, indicating that a population could evolve to a higher mutation rate when beneficial mutations appeared more frequently. As Figure 3 shows, the optimum mutation rate increased from 0.01 to 0.03 as *p_b _*varied from 1% to 10%.

**Figure 4:**
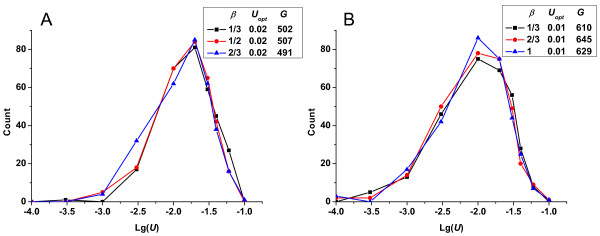
**Change in the frequencies of fixed mutation rates *vs*. lg(*U*)-given different scale parameter *β *of the gamma distribution (*R_u _*= 1)**. (A) In all cases, the conditions were constant: *α *= 0.6, *N *= 10^5^, *p_b _*= 5%, ${\bar{s}}_{b}=0.01$. (B) In all cases, the conditions were constant: *α *= 0.3, *N *= 10^5^, *p_b _*= 1%, ${\bar{s}}_{b}=0.01$.

**Figure 5:**
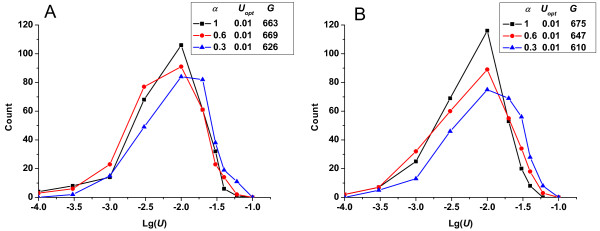
**Change in the frequencies of fixed mutation rates *vs*. lg(*U*)-given different shape parameter *α *of the gamma distribution (*R_u _*= 1)**. (A) In all cases, the conditions were constant: *β *= 1/2, *N *= 10^5^, *p_b _*= 1%, ${\bar{s}}_{b}=0.01$. (B) In all cases, the conditions were constant: *β *= 1/3, *N *= 10^5^, *p_b _*= 1%, ${\bar{s}}_{b}=0.01$.

In Figure 4, we show the change in the frequencies of fixed mutation rates in response to changes in the scale parameter (*β*) of the gamma distribution for describing deleterious mutations effects, while holding the shape parameter and the effects of beneficial mutations constant. The frequencies of the fixed mutation rates varied only slightly and the optimum mutation rate held constant when *β *was varied, although the mean effects of deleterious mutations changed substantially. Evaluation of how the shape parameter (*α*) of the gamma distribution influenced the results was also important. In Figure 5, we show the change in the frequencies of fixed mutation rates in response to changes in *α*, while holding the scale parameter and the effects of beneficial mutations constant. As *α *decreased, the deleterious mutations that had small effects increased while those with large effects decreased, shifting the curve slightly to the right. Varying the shape parameter therefore had more effect on the frequencies of fixed mutation rates than did varying the scale parameter. Nevertheless, the optimum mutation rate remained constant in this case, indicating that deleterious mutations were effectively eliminated by relentless selection and that their effects had little influence on the optimum mutation rate in long-term evolution. Only when the deleterious mutation effects became small enough to compensate for the beneficial mutation effects could the optimum mutation rate evolve to a higher level (data not shown).

**Figure 6:**
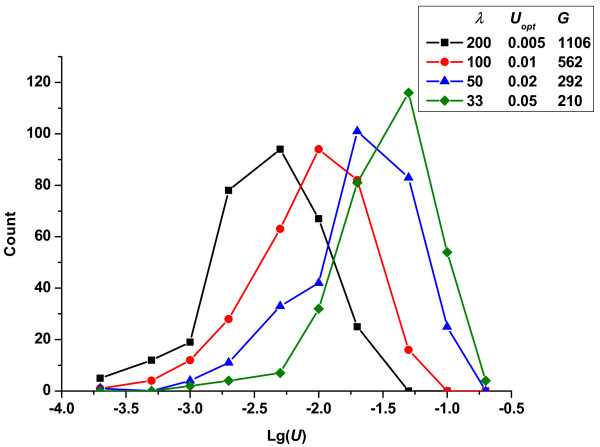
**Change in the frequencies of fixed mutation rates *vs*. lg(*U*)-given different parameter *λ *of the exponential distribution (*R_u _*= 4)**. In all cases, the conditions were constant: *N *= 10^5^, *p_b _*= 3%, ${\bar{s}}_{d}=0.3$.

Finally, in Figure 6, we show the change in the frequencies of fixed mutation rates in response to changes in the parameter (*λ*) of exponential distribution, while holding the effects of deleterious mutations constant. In contrast to the very small impact seen in response to varying the deleterious mutation effects, the beneficial mutation effects contributed significantly to the optimum mutation rate. The curve was substantially shifted to the right with increasing $\bar{s_{b}}$, indicating that populations favored a much higher mutation rate when strong beneficial mutations appeared. The optimum mutation rate changed from 0.005 to 0.05 as $\bar{s_{b}}$ varied from 0.005 to 0.03. In addition, when $\bar{s_{b}}$ increased to 0.03, the mean number of generations required for competition (*G *= 210) shortened sharply compared to ${\bar{s}}_{b}=0.005(G=1106)$. Thus, strongly favored mutations had surprising effects on the competition time, and this type of effect was rarely observed when the other parameters were varied.

## Discussion

In this study, we developed a simulation method based on competition among subpopulations in order to explore the pattern of evolution of mutation rate in large asexual populations. Our simulation results showed that populations tended to form an optimum mutation rate based on their initial configuration. This optimum mutation rate depended on the influx of favorable mutations as well as on their corresponding effects. Below, we first discuss the influence of the initial configuration of the population. We then discuss why beneficial mutations are important in asexual populations. Finally, we compare the present results to previous studies about mutators.

### Influence of the initial configuration of the population

**Figure 7:**
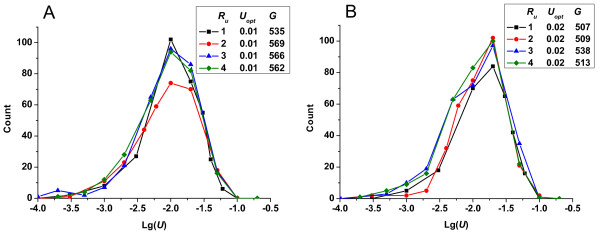
**Change in the frequencies of fixed mutation rates *vs*. lg(*U*)-given different mutation range *R_u_***. (A) In all cases, the conditions were constant: *N *= 10^5^, *p_b _*= 3%, ${\bar{s}}_{d}=0.3$, ${\bar{s}}_{b}=0.01$. (B) In all cases, the conditions were constant: *N *= 10^5^, *p_b _*= 5%, ${\bar{s}}_{d}=0.3$, ${\bar{s}}_{b}=0.01$.

To assess whether different mutation ranges and different initial fitness influenced the optimum mutation rate, we performed new simulations, yielding the following results. In Figure 7, we show the change in the frequencies of fixed mutation rates in response to changes in the range of initial mutation rates assigned to subpopulations. The optimum mutation rate appeared to remain constant for any given initial mutation range, although the exact count was dependent on the interval of mutation rates and thus showed slight differences. The optimum mutation rate depended very little on the particular choice of the mutation range, as long as that range placed the optimum mutation rate at some intermediate value (i.e., lower than the upper limit and higher than the lower limit). In summary, despite the diversity of this mutation range, the optimum mutation rate was essentially determined by population size and mutation effects (Note: even larger orders of ranges gave very rough results and are not shown).

**Figure 8:**
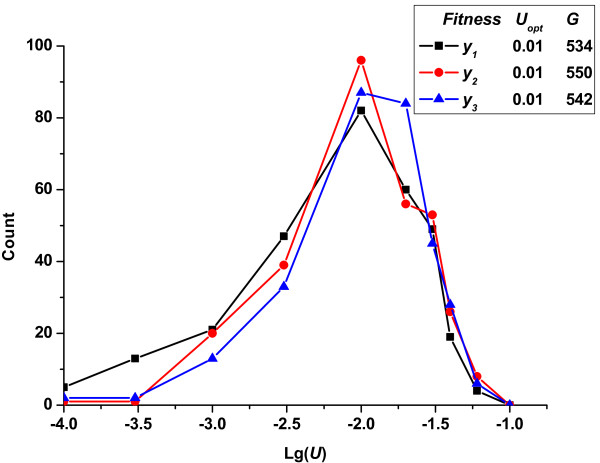
**Change in the frequencies of fixed mutation rates *vs*. lg(*U*)-given different initial individual fitness (*R_u _*= 1)**. *y_1 _*represents normal distribution with mean μ = 0.5 and variance σ^2 ^= 0.1; *y_2 _*represents gamma distribution with α = 20, β = 20; and *y_3 _*represents the initial fitness of all individuals equal to a unified value 1.0. In all cases, the conditions were constant: *N *= 10^5^, *p_b _*= 3%, ${\bar{s}}_{d}=0.3$, ${\bar{s}}_{b}=0.01$.

We also assigned rugged initial fitness to replace the assumption that all individuals had unified initial fitness value of 1.0. In Figure 8, we show the change in frequencies of fixed mutation rate given that the initial fitness of all individuals followed a normal or a gamma distribution. The results confirmed that the optimum mutation rate was little influenced by the initial fitness of individuals.

**Figure 9:**
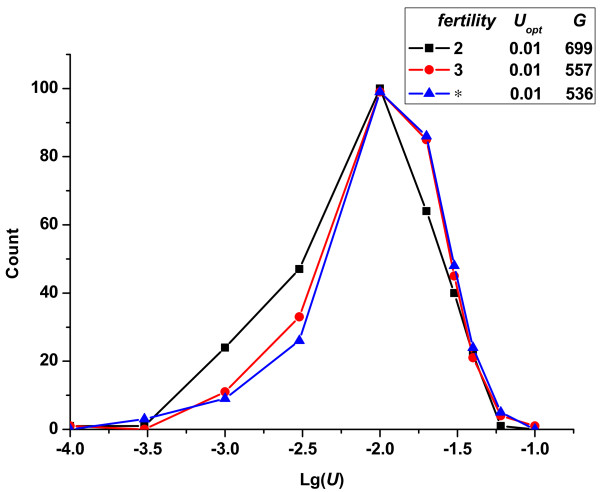
**Change in the frequencies of fixed mutation rates *vs*. lg(*U*)-given different fertility limitation (*R_u _*= 1)**. '*' represents no limitation in fertility. In all cases, the conditions were constant: *N *= 10^5^, *p_b _*= 3%, ${\bar{s}}_{d}=0.3$, ${\bar{s}}_{b}=0.01$.

Finally, we show the influence of organism's fertility limitation on the optimum mutation rate in Figure 9. Fertility is defined as the upper limit on the number of offspring per individual per generation. Because this limitation might slow down the spread speed of beneficial mutations, we assumed different fertility of the population to test that whether such limitation would cause a lower optimum mutation rate. Nevertheless, the results in Figure 9 showed that a limitation in fertility led to no difference in the corresponding optimum mutation rate. Taking the influence of random factors in simulation studies into account, such small differences in frequencies of fixed mutation rates could be neglected. However, a long time would be needed to complete one competition process when fertility was limited (insert box of Figure 9). Although the fertility limitation decelerated the adaptation process of an organism indeed, it had little effect on the optimum mutation rate. This might provide an additional explanation for Drake's observation of constant genome mutation rates across DNA-based microbes, despite their different fertility mechanisms.

To summarize, the initial configuration of the population had little influence on the optimum mutation rate, demonstrating the robustness of the developed method based on competition among subpopulations.

### Beneficial mutations are crucial in shaping the optimum mutation rates

In this study, we have made three assumptions about mutation effects: first, the mean effects of deleterious mutations are much larger than those of beneficial ones; second, beneficial mutation effects are exponentially distributed; and finally, deleterious mutations effects follow a gamma distribution. However, our simulation results hinge mainly on the first two assumptions. The first assumption is likely to be biologically realistic in many cases, although surely not universally true. Indeed, theory analysis [27,28] and empirical research (see Table 1) have shown that the mean effects of deleterious mutations are greater than those of beneficial ones. In addition, we assumed that the effects of beneficial mutations followed an exponential distribution, which has good theoretical [22-24] and empirical support [32,35,37]. Therefore, the exponential distribution seems a reasonable choice. As for the third assumption, we do not yet have a good understanding of the distribution of deleterious mutation effects due to their complexity. However, the effects of deleterious mutations had little influence on the optimum mutation rate as long as not producing an excessive amount of slightly deleterious mutations. If the mean effects of deleterious mutations was too small to counteract the beneficial mutation effects (e.g., $\bar{s_{d}}$ is smaller than 0.01), the optimum mutation rate might reach a higher value than the one presented.

In general, organisms are well adapted to their living environments, so only a few changes lead to fitness increases and these beneficial mutations have very small effects [32,34,35,37]. In a recent study, Cowperthwaite et al [38] used an *in silico *system to show that beneficial mutations with small effects have always existed in the process of evolution. Although beneficial mutations are much rarer compared to deleterious mutations, they supply the driving force for adaptive evolution and contribute to survival of populations in tough environments [39]. As shown in our results, an increase in either the fraction or the effects of beneficial mutations led to a parallel increase in the optimum mutation rate. It is established that in asexual populations, two problems affect the adaptation: clonal interference and multiple mutations; clonal interference causes beneficial mutations in different genetic backgrounds compete with one another, while multiple mutations in the same background could assist each other's spread toward fixation [40-44]. How the both factors determine the rate at which asexual population evolve has been investigated in recent studies and their actions are related to influx of beneficial mutations, including their fraction (*p_b_*) and effects (*s_b_*) [41,43].

If the fraction of beneficial mutations (*p_b_*) is relatively high, the clonal interference becomes important. However, in this case, there will also be more chances for multiple beneficial mutations to occur in the same genetic background. Whenever clonal interference is important, so are multiple mutations. As Desai and Fisher showed, evolution in asexual budding yeast populations was dominated by the accumulation of multiple mutations of moderate effect [43]. Individuals that carry multiple beneficial mutations probably have higher fitness than those with one original beneficial mutation. Thus, a subpopulation with a higher mutation rate could benefit from more multiple beneficial mutations, as Figure 3 shown.

On the other hand, if the effects of the majority of new arising beneficial mutations (*s_b_*) are small, these mutations need more generations to be fixed and remain at low frequency in the population for quite a long time [45]. This provides a sufficient chance for competing mutations to occur in the ensuring generations, causing the beneficial mutation with small effects to be wasted [46]. By contrast, if the beneficial mutation effects increase (i.e., strong beneficial mutation appears), natural selection increases their fixation probability and shortens its fixation time, thus reducing the effect of clonal interference [42,45,47]. This may explain why competition time among subpopulations was significantly shortened when *s_b _*increased. As Wilke pointed out, in the presence of clonal interference, adaptation speed in asexuals still continued to grow with the mean beneficial mutation effects [21], although in a decelerating way. Therefore, reduction in the effect of clonal interference due to increasing *s_b _*may further increase the adaptation rate of populations considerably. In this case, the population favored a much higher mutation rate. Our simulation results indicated that if strong beneficial mutations (${\bar{s}}_{b}=0.03$) were produced, the population would evolve to a much higher optimum mutation rate (*U*_opt _= 0.05).

This might provide an alternate explanation for why viruses are capable of evolving to a much higher mutation rate [48] under the influence of the responding rate of immune systems [49]. To survive in extremely stressful environments, the virus populations must evolve more beneficial mutations with large effects.

### Selection on mutation rate in asexual populations

The action of selection on mutation rate can be classified as either direct or indirect: direct action is dependent on the effects of modifier alleles on fitness, while indirect action is dependent on the "linkage disequilibrium" between modifier alleles and alleles at other loci affecting fitness [19]. Strong effect modifiers that increase mutation rates are called mutators [12,50]. Considerable theoretical literature exists on the evolution of mutation rates based on the evolutionary fate of mutators [11,12,51]. For instance, Ander and Godelle [12] elucidated the fate of modifiers of mutation rates and obtained three results: first, when adaptation has a significant role, a strong-effect mutator will emerge. Second, the modifier with large effects is likely to appear only when the fitness cost of deleterious mutations is very weak. Third, in small populations, the mutation rate is always blocked at a lower level. In the present study, the optimum mutation rate increased with the effects of beneficial mutations, in agreement with their first result. We also pointed out that effects of deleterious mutations had little influence on the optimum mutation rate unless an excessive number of slightly deleterious mutations were produced, in agreement with their second result. Finally, in our study, when everything else being equal, large populations would evolve to higher optimum mutation rates, in agreement with their third result.

Nevertheless, it should be noticed that in all of the previous studies, high genome mutation rates were generally disfavored in asexual populations except when organisms were under extreme conditions. Gerrish et al. suggested that in the case of complete linkage, the mutation rate would continue to increase until it reached an intolerable level and then lead to organism extinction, rather than elevate without a ceiling [51]. The intuitive picture is that selection would drive mutation rate toward a maximum value when beneficial mutations are occurring [19]. However, as Gerrish et al. pointed out that natural selection, although very robust, is a short-sighted process that favors individuals with immediate fitness benefits. A mutator could get such immediate profits from a beneficial mutation, whereas its action might be weakened by the eventual increase in deleterious mutations.

Other studies involving modifiers also suggested that even if a high mutation rate increased the rate of adaptation in the short term, due to deleterious mutations, selection would be likely to decrease the mutation rate in the long term evolution [19,52-54]. Thus the evolution of mutation rate in an asexual system would yield an optimum compromise between deleterious and beneficial mutations, as the present study indicated.

## Conclusions

Based on competition among subpopulations with different mutation rates, we investigated the evolution of mutation rates in finite asexual populations. The efficiency of natural selection on mutation rate was shown to depend on population size and mutation effects. Large populations tend to have high mutation rates. The optimum rate is also the result of a balance between two opposing forces: a decreasing rate caused by deleterious mutations and adaptation caused by beneficial mutations. However, the influx of favorable mutations is the critical factor and largely determines the optimum mutation rate in large asexual populations. Contrary to our intuition, the effects of deleterious mutations have little impact on this rate as long as there is no an abundance of deleterious mutations with tiny effects. We hope this simulation method and these findings provide useful inspiration for further modeling of the evolution of mutation rates in asexual populations.

## Authors' contributions

XJ carried out the simulation experiments, analyzed the data, and drafted the manuscript. BM and ZH participated in writing the computer programs. MZ and XW proposed important suggestions for conceiving the simulation process. ST conceived of the study and supported the research. All authors have read and approved the final version of the manuscript.
